# The Practitioner's Handbook of Treatment

**Published:** 1898-09

**Authors:** 


**The Practitioner's Handbook of Treatment.** By the late J. MILNER FOTHERGILL, M.D. Fourth Edition. Edited and in great part rewritten by William Murrell, M.D. Pp. xviii., 688. London: Macmillan and Co., Limited. 1897.

In the preface to the first edition this book was announced as "an attempt of original character to explain the *rationale* of our therapeutic measures," and not as an imperfect practice of physic. This is indeed the key-note to the book, which attained a wide popularity partly from the author's charming style and partly from the constant references to physiological principles with which he rendered interesting the details of treatment. In another respect it had a great value to the young practitioner, for Dr. Fothergill laid great stress on the recognition of the rational symptoms of disease as distinct from the physical signs, and taught the necessity of noting the *facies morbi*, the appearance and surroundings of the patient. The student has too often been obliged to gather this knowledge from the experience of his errors, and the warnings have been therefore of the utmost utility. The numberless practical hints of an old practitioner scattered through the book corrected defects of a student trained in hospital work alone, and the many prescriptions given taught a much needed skill in combining drugs. In short, it was unfortunate that such a work should decay from sheer age, and Dr. Murrell has well revived its beauties and extended its useful life by his careful revision. It remains one of the most stimulating and practical books which we have, though one which presupposes a sound knowledge of ordinary clinical text-books. Thus in the charming chapter on body heat and fever much insight is given into the physiology of disease and the means of treating patients in a febrile state with advantage: but we should like fuller directions on cold baths in typhoid fever, excellent as the remarks on the subject are; many of us, too, will hesitate to endorse Dr. Murrell's praise of the safety of veratrum, aconite, and pilocarpine. The action of the latter drug he says can be stopped instantly by a minute dose of atropine, but it would be well if the reader were warned of those conditions which have led to serious accidents and which contra-indicate its use. It is not clear what the benefit of using carbolised oil for the skin of scarlatina patients can be; for the disinfectant powers of the carbolic are reduced to a minimum, while the chances of absorption remain the same. The chapter on the digestive system seems to require considerable revision to bring it into line with modern knowledge. The reader is apt to forget that careful treatment of dyspepsia, flatulence, and biliousness must now be based on the enormously increased physiological and pathological knowledge of the last ten years, and the few pages devoted to stomach affections may foster the idea that its various well-defined diseases may be treated by a routine of bismuth and aperients. The skilful physician indeed can pick up useful hints, but to the man whose usual diagnosis is flatulence the section is a dangerous one. In diabetes exercise is recommended; but the terrible risk of over-exertion or shock, as well as that from a chill needs emphasising. Similarly, in rheumatism we find an excellent account of present treatment of the acute disease; but a stronger caution should be given as to the probability of relapse if salicylates are stopped too soon, or if the patient gets up. We are all apt to forget that salicylic treatment, while it quickly removes all the symptoms, has not removed the tendency to relapse if the drug is stopped before the natural course of the disease is over. Opinions too will differ as to the recommendation of digitalis at once if the mitral valve is injured (p. 277). Indeed it does not seem self-evident or good English to say that "no amount of dilatation will render the mitral valve incompetent, if its valvular vela are not restrained by pathological connective tissue from themselves stretching along with the dilated ventricle." It might be argued that incompetence occurs commonly enough in the dilatation of chlorosis where the tissues are normal.

The difficulties in revising a somewhat discursive work are doubtless very great, and our thanks are certainly due to Dr. Murrell for an arduous labour. He has preserved much of the charm of the book, and enriched it from his ripe stores of knowledge with great success; yet it must not be regarded as a treatise on the practice of physic, but as an explanation of the reasons for certain methods of treatment with much sound advice on the examination and management of patients.

